# Toxicity and Functional Tissue Responses of Two Freshwater Fish after Exposure to Polystyrene Microplastics

**DOI:** 10.3390/toxics9110289

**Published:** 2021-11-02

**Authors:** Martha Kaloyianni, Dimitra C. Bobori, Despoina Xanthopoulou, Glykeria Malioufa, Ioannis Sampsonidis, Stavros Kalogiannis, Konstantinos Feidantsis, Georgia Kastrinaki, Anastasia Dimitriadi, George Koumoundouros, Dimitra A. Lambropoulou, George Z. Kyzas, Dimitrios N. Bikiaris

**Affiliations:** 1 Laboratory of Animal Physiology, Department of Zoology, School of Biology, Aristotle University of Thessaloniki, 54124 Thessaloniki, Greece; kaloyian@bio.auth.gr (M.K.); despxant@bio.auth.gr (D.X.); mglykeri@bio.auth.gr (G.M.); kfeidant@bio.auth.gr (K.F.); 2 Laboratory of Ichthyology, Department of Zoology, School of Biology, Aristotle University of Thessaloniki, 54124 Thessaloniki, Greece; 3 Department of Nutritional Sciences and Dietetics, International Hellenic University, 57001 Thessaloniki, Greece; isampson@nutr.teithe.gr (I.S.); kalogian@nutr.teithe.gr (S.K.); 4 Laboratory of Inorganic Materials, CERTH/CPERI, 57001 Thessaloniki, Greece; georgiak@cperi.certh.gr; 5 Biology Department, University of Crete, 70013 Herakleion, Greece; adimitriadibio@gmail.com (A.D.); gkoumound@uoc.gr (G.K.); 6 Laboratory of Environmental Pollution Control, Department of Chemistry, Aristotle University of Thessaloniki, 54124 Thessaloniki, Greece; dlambro@chem.auth.gr; 7 Department of Chemistry, International Hellenic University, 65404 Kavala, Greece; kyzas@chem.ihu.gr; 8 Laboratory of Polymer Chemistry and Technology, Department of Chemistry, Aristotle University of Thessaloniki, 54124 Thessaloniki, Greece

**Keywords:** polystyrene, microplastics, *Danio rerio*, *Perca fluviatilis*, gills, liver, metabolomics, oxidative stress biomarkers

## Abstract

Microplastics (MPs)’ ingestion has been demonstrated in several aquatic organisms. This process may facilitate the hydrophobic waterborne pollutants or chemical additives transfer to biota. In the present study the suitability of a battery of biomarkers on oxidative stress, physiology, tissue function and metabolic profile was investigated for the early detection of adverse effects of 21-day exposure to polystyrene microplastics (PS-MPs, sized 5–12 μm) in the liver and gills of zebrafish *Danio rerio* and perch, *Perca fluviatilis*, both of which are freshwater fish species. An optical volume map representation of the zebrafish gill by Raman spectroscopy depicted 5 μm diameter PS-MP dispersed in the gill tissue. Concentrations of PS-MPs close to the EC_50_ of each fish affected fish physiology in all tissues studied. Increased levels of biomarkers of oxidative damage in exposed fish in relation to controls were observed, as well as activation of apoptosis and autophagy processes. Malondialdehyde (MDA), protein carbonyls and DNA damage responses differed with regard to the sensitivity of each tissue of each fish. In the toxicity cascade gills seemed to be more liable to respond to PS-MPs than liver for the majority of the parameters measured. DNA damage was the most susceptible biomarker exhibiting greater response in the liver of both species. The interaction between MPs and cellular components provoked metabolic alterations in the tissues studied, affecting mainly amino acids, nitrogen and energy metabolism. Toxicity was species and tissue specific, with specific biomarkers responding differently in gills and in liver. The fish species that seemed to be more susceptible to MPs at the conditions studied, was *P. fluviatilis* compared to *D. rerio*. The current findings add to a holistic approach for the identification of small sized PS-MPs’ biological effects in fish, thus aiming to provide evidence regarding PS-MPs’ environmental impact on wild fish populations and food safety and adequacy.

## 1. Introduction

Polystyrene is a thermoplastic synthetic polymer with appropriate thermal and mechanical properties and can be used in many applications even though it is characterised as a hard and brittle material. The worldwide production of polystyrene was close to 15.61 million metric tons in 2019 and it is estimated to be stabilized at these levels for the next years, while it covers about 5–6% of the global plastic production [1]. It is a colourless transparent polymer used in household applications, electronics, packaging, isolation foams, single used items like disposable cutlery, etc. [2]. Due to the lightness of most of its products, it is very difficult to recycle and unfortunately the majority of it is disposed of in the environment, contributing significantly to the formation of microplastics (MPs). These MPs are plastic fragments with sizes less than 5 mm and can cause serious health problems for all living organisms including human beings [3,4,5,6,7,8].

Research on freshwater ecosystems has started to gain attention since rivers act as the main pathways for plastic transport to seas [9,10]. In a recent literature search, only 16.2% of the published papers concerning microplastic pollution, were focused on freshwater environments [11].

It is clear, independently from the ecosystem concerned (marine/freshwater or even terrestrial), that plastic debris are being broken down by numerous procedures, such as UV degradation, oxidation and erosion, resulting in smaller fragments, with a vast range in sizes [12,13]. Ingestion of MPs, which may facilitate the hydrophobic waterborne pollutants’ or chemical additives’ transfer to biota, is a process which has been demonstrated in a range of aquatic organisms belonging to different taxonomic groups, including invertebrates and vertebrates such as, amphibians [14], fish, sea turtles, seabirds and marine mammals [15,16,17,18,19,20,21].

The information concerning MPs biological effects on freshwater organisms is to date much limited [22,23,24,25]. Among others, studies have already demonstrated the existence of plastic chemicals in fish tissues [26,27]. This evidence has alarmed researchers to examine the transfer of MPs through trophic food chains and study the impacts of MPs on biota that constitute food supply for humans [27,28,29]. This increase in awareness about the MPs ecological impacts is owned to their small size that enables absorption by biota and as a result aggregation in the food chain occurs; in addition, MPs can assimilate contaminants on their surfaces [30], exerting thus additive effects to biota [31,32] and becoming more resistant to deterioration by microorganisms [33].

Fish consume plastic fragments accidentally, usually mixed with their natural prey [12,13]. Micro and nano plastic particles can be transferred to living cells through the lymphatic or circulatory system. This results to MPs’ dispersion in the whole body and the induction of severe effects such as decreased feeding activity [34], impeded growth and development [35,36,37], endocrine disruption [38] and energy destruction [39], oxidative stress (for a review see [40], immunity and neurotransmission disorders [40,41], genotoxicity [42] and even mortality [37]. Internalization of MPs can also occur after they are adhered to fish skin or other tissues, such as gills [26]. Accordingly, MPs are concentrated through the circulation mainly in the gastrointestinal track [43], causing further histopathological alterations in the intestine, physical damage, changes in fish behavior, while translocation to liver tissue may occur, inducing a lot of unfavorable effects [44,45].

Several biomarkers have so far been suggested and examined in laboratory experimental exposure (via water or food) on fish thus offering a first set of implements that can be used to quantify the consequences that chemicals exert [46,47,48]. Among them oxidative stress biomarkers (as usually directly measured through free radical production, protein oxidation increase, lipid peroxidation), genotoxicity (DNA damage increase) and metabolic biomarkers (definition of differentiations in metabolic pathways) are considered as the most commonly used in such studies [49,50,51,52].

In the present study the effects of PS-MPs sized 5–12 μm on two tissues, liver and gills, of two freshwater fish species, zebrafish *Danio rerio* and perch, *Perca fluviatilis*, are monitored. The comparison of the effects on multiple levels on the two vital tissues in two quite distinct fish species will improve our perception of the biochemical and physiological mechanisms implicated in MPs toxicity in order to assess the effects on fish health and population dynamics as well as the suitability of a battery of biomarkers for the early detection of adverse effects of MPs on the environment. Gills were selected as an appropriate marker for evaluating toxicity effects of PS-MPs since they are highly perfused, while liver tissue acts as a detoxifying organ of contaminants entering fish body [53,54], both tissues used in estimating the adverse pollutants effects imposed to organisms [55]. The ultimate goal of our research is to suggest the most suitable fish species, tissue and set of biomarkers to be audited with regards to PS-MPs pollution. Both fish species have been used in biomonitoring studies against pollutants previously [36,46,47,56,57]. To our knowledge, it is the first time that the effects of PS-MPs on the gills and liver of two freshwater fish are investigated by using the combined metabolomic and toxicity methods.

## 2. Materials and Methods

### 2.1. Synthesis and Characterization of Polystyrene Microplastics (PS-MPs)

The preparation of PS microparticles was described in our previous work using the water oil emulsion technique [52]. As found by scanning electron microscopy, spherical PS-MPs with and mean average diameter about 8 ± 3 μm were prepared.

### 2.2. Characterization of Polystyrene Microplastics (PS-MPs) in Fish Parts

Regarding the Fourier transform infrared microscopy, the control and exposed gill and liver were pestled in a mortar with 0.24 g spectroscopic grade KBr powder and dehydrated in room temperature in a desiccator until any moisture was removed. The mixture was then pressed to a pellet form under 6 tons pressure. The microspheres were mixed with KBr in 1% wt for the PS pellet formation. A Jasco spectrometer (Jasco FTIR-6700, Tokyo, Japan) was employed for pellet measurement. In the spectral range of 4000–400 cm^−1^ seventy scans with a resolution of 4 cm^−1^ were collected in the absorbance mode. A 19- point Savitzky–Golay algorithm calculated the second derivative absorption spectrum by the Spectra Manager 2.15.12 software (Jasco Corporation, Tokyo, Japan) by a 17-point Savitzky–Golay algorithm according to relative processing [58]. The HR (high resolution) volume mapping video was calculated by a micro-Raman in Via Instrument coupled with a solid state 532 cm^−1^ laser and ×10 Leica lens. The zebrafish gill was dehydrated in a desiccator chamber for 48 h prior to measurement; the analyzed area was 255 × 192 × 10 μm and the step resolution was 1 μm. Next, 517,400 spectra were collected and analyzed by the Wire 5.3 software for the digital construction of the HR volume mapping video (Supplementary Material). The color map was created in respect to the intensity of the spectrum at the characteristic PS peak at 995 cm^−1^.

### 2.3. Fish Husbandry

Adult specimens of zebrafish (*D. rerio*, ZF WT 2 F10, Wageningen Agricultural University, Wageningen, The Netherlands) were provided by the Department of Biology of the University of Crete. Fish had (mean ± SD) total length and body weight equal to 33.5 ± 2.7 mm and 0.25 ± 0.07 g respectively, aged 6 months, while no sex separation was conducted. All specimens were acclimatized in aerated fish tanks (3 individuals per liter) with water pump circulation through filters, under 14:10 light:dark cycle, at 28 ± 0.2 °C temperature, 8.57 ± 0.23 pH, 8.94 ± 0.14 mg L^−1^ dissolved oxygen, 560 ± 37.9 μS cm^−1^ conductivity and 0.2 ± 0.06 psu salinity.

Specimens of both sexes of wild fish species *P. fluviatilis* (mean ± SD total length 13.5 ± 3.08 cm and body weight 27.2 ± 19.67 g) were provided alive from Lake Volvi (Northern Greece) by a commercial fisherman, while no sex separation was conducted. Immediately they were placed in aquariums of 150 L water volume (six individuals per liter), equipped with circulated pumps for filtering and cleaning water. Fish were maintained at well aerated water of 19.2 ± 0.94 °C, pH 7.4 ± 0.92, dissolved oxygen concentration 10.6 ± 0.33 mg L^−1^, conductivity 609 ± 41.1 μS cm^−1^ and salinity 0.5 ± 0.29 psu. The photoperiod was adjusted to a 14:10 light:dark cycle.

During the 7-day acclimatization period zebrafish were fed once per day with commercial flakes (Cichlid Omni Flakes, Ocean Nutrition Europe, Essen, Belgium) while fish excrement in aquariums was removed manually, every day with a net. Perch were acclimatized for 25 days, their feeding was based on commercial dry shrimps *Gammarus pulex* (Tropical company, Dover, DE, USA) and applied once per day.

### 2.4. Ethical Statement

All the experimental procedures involving handling and exposure of fish were performed in accordance with Greek (PD 56/2013) and EU (Directive 63/2010) legislation for animal experimentation and welfare. All protocols were approved by the Animal Care Committee of the Biology Department of the University of Crete (Permit Number: 285586(2020)).

### 2.5. Food Preparation

Food was prepared following the procedure described previously [46]. In brief, an amount of PS-MPs (5–12 μm in size) in water suspension was incorporated into commercial powder fish food (different for each species as given above), to obtain the target concentrations (see below). Subsequently the mixture was well homogenized and dried in oven for about 2 h at 50 °C. Food for control groups was prepared following the same procedure as in previous study [52], omitting the addition of MPs. The heating of food could degrade food nutrient composition and possibly affect fish sensitivity to contaminants. However, since the control food was treated the same, this may not affect our results. In all experiments each fish was fed once per day with food corresponding to 3% of its wet weight [59], that is equivalent to 0.0075 g and 0.85 g for zebrafish and perch respectively. Fish were inspected during feeding, ensuring thus that all food was consumed.

### 2.6. EC_50_ Estimation (1st Experimental Design)

EC_50_ value has been largely employed as a potent and reliable index for the estimation of the sublethal concentration of various toxicants in several studies of our group, in aquatic animals and snails [46,47].

For the estimation of EC_50_ in zebrafish, 24 individuals were divided into three groups (eight individuals per group, each group in different tank). Each group was fed, once per day, with food containing nominal concentrations of 1, 50 and 100 mg PS-MPs per g dry weight for 21 days. This period is within the range of 4 h to 2 months used by several researchers as an exposure time in laboratory experiments to assess the toxicological effects of MPs in fish (see review by Phuong et al. [60]) and was selected to depict the associated toxicity mechanisms in organisms corresponded to highly pronounced contaminant stress. An additional group of eight zebrafish, fed with commercial food without PS-MPs addition under the same conditions, served as control. After the end of exposure time, blood samples were collected from the tail and centrifuged at 3000× *g* for 10 min at 4 °C. Specifically, tail was dissected, and blood removed from the dorsal aortic canula was collected in heparinized Eppendorf tubes. Subsequently the 3-(4,5-dimethylthiazol-2-yl)-2,5-diphenyltetrazolium bromide (MTT) levels were measured [61] in the blood samples in order to assess mitochondrial redox capacity. The mean values of MTT obtained from eight zebrafish were calculated for each feeding concentration and the EC_50_ value was estimated using the SPSS software. The EC_50_ value for zebrafish was calculated at 10 mg of PS-MPs g^−1^ of dry food and this concentration was used for preparing zebrafish food for the consecutive in vivo exposure experiments.

For the estimation of EC_50_ in perch, a similar experimental procedure as that described before was followed. In specific, nine individuals were divided into three groups (three individuals per group, per tank). In addition, three specimens were used as the control group. Each group was fed, once per day, with food containing nominal concentrations of 1, 50 and 100 mg PS-MPs g^−1^ dry weight for 21 days. After the end of exposure time, the destabilization of the hemocyte’s lysosomal membranes was assayed in the blood samples by the Neutral Red Retention Time (NRRT) assay [62] as modified by Dailianis et al. [63]. The EC_50_ value for perch was calculated at 134 mg of PS-MPs g^−1^ of dry food, which was used in the subsequent experiment.

MTT and NRRT uptake assays are both cellular biomarkers. MTT represents mitochondrial redox capacity and NRRT provides information on the lysosomal activity of the cells. Since zebrafish blood volume was much less in relation to that of perch, MTT assay was preferred over NRRT due to the less blood required for conducting the assay.

Since data about the environmental concentrations of PS-MPs in freshwater aquatic ecosystems is limited [42], our experiments were designed mainly to assess the response of both fish species under sublethal PS-MPs concentrations, simulating a highly pessimistic pollution scenario. Our approach was to provoke accelerated effects on fish exposed to higher concentrations than those reported in the field. The use of higher concentrations than those found in the field, in order to depict the associated MPs toxicity mechanisms is very common in such laboratory studies, thus, the ingestion and toxic effects observed in organisms correspond to highly pronounced contaminant stress (see review by Phuong et al. [60]). In accordance with the concentrations of MPs used in the present study, the use of 40 mg g^−1^ has been reported for zebrafish experiments [64], while Solomando et al. [65] exposed *Sparus aurata* to 100 mg MPs g^−1^.

### 2.7. Fish Feeding Exposure to PS-MPs (2nd Experimental Design)

Experiments for assessing the response of both fish to PS-MPs were run in parallel. Zebrafish control (*n* = 30–10 individuals per aquarium) and exposed individuals (*n* = 30, 10 individuals per aquarium) were kept in aquariums of 30 L with circulated water, external oxygenation and the same conditions as in the acclimatization stage. Fish were fed once per day with food containing 10 mg PS-MPs g^−1^ of dry food for 21 days while control animals were fed with food without added MPs. Accordingly, perch specimens were divided into two groups, the control (*n* = 6, 2 individuals per aquarium) and the experimental group (*n* = 6, 2 individuals per aquarium) and kept in aquariums under the same conditions as those previously described. Fish were fed once per day with pellets containing 134 mg PS-MPs g^−1^ of dry food for 21 days, except the control group which was fed with commercial food for percids, without the addition of PS-MPs. During the treatment period the water in aquariums was kept at a constant volume by adding the appropriate quantity of water. Νo fish mortality was observed, either in the control or the exposed groups for both species.

### 2.8. Tissue Sampling

After the treatment period, control and exposure fish of both species were anaesthetized (zebrafish in cold water and perch in ethanol clove oil diluted in water), immediately placed on ice and blood samples were taken from the caudal area and placed in tubes with heparin. Gills and liver tissues were consequently extracted from both fish, placed in tubes and stored at −30 °C (for approximately 1 month) until further analyses and were used for the estimation of lipid peroxidation, protein carbonylation, DNA damage, ubiquitin conjugates, autophagic and apoptotic processes and metabolomics analysis.

### 2.9. Molecular and Biochemical Analyses

All analyses described below were assessed in the liver and gills of the whole population (*n* = 30 individuals of *Danio rerio*) divided in 3 pools of 10 fish and each pool was analyzed separately (*n* = 3 pools). For *Perca fluviatilis n* = 6 individuals per experimental condition (control and exposure) were used and the tissues of 2 fish were pooled and analyzed together forming 3 different pools.

The estimation of lipid peroxidation in gills and liver tissues followed the method described by Niehaus and Samuelsson [66]. Frozen tissues were immediately homogenized in 50 mmol L^−1^ phosphate buffer (pH 7.4). The homogenate was then centrifuged (2000× *g*, 4 °C, 15 min), and immediately 250 μL of 20% TCA and 500 μL of 0.67% thiobarbituric acid were added in 250 μL of supernatant. The mixture was vortexed, boiled for 60 min, and cooled at room temperature. Thereafter, 2 mL of butanol was added and the mixture was again centrifuged (3000× *g*, 15 min). The results are expressed as nmol malondialdehyde (MDA) per mg protein (protein concentration was determined by using the BioRad protein assay), since one of the terminal products of lipid peroxidation is MDA. The concentration of MDA was detected at 535 nm (ε = 156 mM^−1^  cm^−1^) [67].

The content of protein carbonylation (PCC) was determined according to Buss et al. [68] and Alamdari et al. [69]. However, the procedure followed herein is modified since protein samples are first absorbed to an ELISA 96 well plate through overnight incubation at 4 °C) and then react with 2,4-dinitrophenylhydrazine (DNPH). Quantification of PCC was based on a standard curve produced by measuring at 450 nm 5 μg bovine serum albumin (BSA) instead of 60 μg proposed by Buss et al. [68]. Forms of reduced and oxidized BSA were employed for the creation of a standard curve [69]). The PCC content was quantified according to the standard curve of BSA (y = 1.4033x + 0.002), R^2^ = 0.9916, bovine serum albumin concentrations used were 0–0.25 μΜ. The results were expressed as nmol carbonyl groups mg^−1^ of protein.

The levels of ubiquitinated proteins and caspases conjugates in gills and liver of both fish species were quantified using well established methodology. Frozen tissues were immediately homogenized in 3 mL g^−1^ of cold lysis buffer (20 mM β-glycerophosphate, 50 mM NaF, 2 mM EDTA, 20 mM Hepes, 0.2 mM Na_3_VO_4_, 10 mM benzamidine, pH 7, 200 μM leupeptin, 10 μΜ trans-epoxy succinyl-Lleucylamido-(4-guanidino)butane, 5 mM dithiotheitol, 300 μΜ phenyl methylsulfonyl fluoride (PMSF), 50 μg mL^−1^ pepstatin and 1% *v*/*v* Triton X-100), and extracted on ice for 30 min. Samples were centrifuged (10,000× *g*, 10 min, 4 °C) and the supernatant was boiled with 0.33 volumes of SDS/PAGE sample buffer (330 mM Tris-HCl, 13% *v*/*v* glycerol, 133 mM DTT, 10% *w*/*v* SDS, 0.2% *w*/*v* bromophenol blue). Protein concentration was determined by using the BioRad protein assay. Thereafter, samples were immersed in a nitrocellulose membrane (0.45 μm, Schleicher & Schuell, Stockbridge, GA, USA), set in a dot blot (BioRad, Hercules, CA, USA) vacuum apparatus. As antibodies were used a polyclonal anti-ubiquitin rabbit antibody (Cat. No. 3936, Cell Signaling, Beverly, MA, USA) and a monoclonal anti-cleaved caspase rabbit antibody (Cat. No.8698 Cell Signaling, Beverly, MA, USA). Thereafter, nitrocellulose membranes were washed with TBST (3 × 5 min). Then, an 1 h incubation with a horseradish peroxidase linked secondary antibody (7074, 7076, Cell Signaling, Beverly, MA, USA) followed and membranes were washed with TBST (3 × 5 min). The dots were detected using enhanced chemiluminescence (Chemicon) on Fuji Medical X-ray film and quantified by densitometry scanning laser (GelPro Analyzer Software, GraphPad, San Diego, CA, USA).

The method modified by Dailianis et al. [70] was applied for the estimation of DNA damage in both fish tissues examined. After gill and liver cells were treated with collagenase, DNA lysis and electrophoresis under neutral conditions, and DNA staining with acridine orange [71], the presence of comets was examined and counted under fluorescent microscope (Olympus CKX41) following the criteria of Ritter and Knebel [72]. Detailed description of the procedure of DNA damage is referred by Dimitriadi et al. [52]. In brief, six slides per pool (zebrafish) and six slides per individual (perch) were measured, in order to represent technical replicates. Randomly selected 100 cells were scored from each slide (Tritek Cometscore^TM^ 1.5, TriTek Corporation, Wilmington, DE, USA). Moreover, PS-MPs free cells were exposed to H_2_O_2_ (1 μΜ) in order to verify the comet assay method electrophoresis conditions as well as the genotoxicity of H_2_O_2_ (positive control) as previously published [73]. The results are expressed as % DNA in tail (percentage of DNA in comet tail). % DNA in tail and Olive moment in positive control data (1 μΜ H_2_O_2_) were 28.3 ± 5.2 and 40 ± 6.3, respectively. The results are expressed as percentage of DNA in tail (% DNA in tail).

Autophagic and apoptotic indicators were quantified by SDS/PAGE immunoblot techniques. Frozen gill and liver tissue samples from control and PS-MPs treated individuals of both fish species were homogenized in 3 mL g^−1^ of cold lysis buffer (20 mM β-glycerophosphate, 50 mM NaF, 2 mM EDTA, 20 mM Hepes, 0.2 mM Na_3_VO_4_, 10 mM benzamidine, pH 7, 200 μM leupeptin, 10 μΜ trans-epoxy succinyl-Lleucylamido-(4-guanidino)butane, 5 mM dithiotheitol, 300 μΜ phenyl methylsulfonyl fluoride (PMSF), 50 μg mL^−1^ pepstatin, 1% *v*/*v* Triton X-100), and extracted on ice for 30 min. Samples were centrifuged (10,000× *g*, 10 min, 4 °C) and the supernatant was boiled with 0.33 volumes of SDS/PAGE sample buffer (330 mM Tris-HCl, 13% *v*/*v* glycerol, 133 mM DTT, 10% *w*/*v* SDS, 0.2% *w*/*v* bromophenol blue). Protein concentration was determined by using the BioRad protein assay. Thereafter, equivalent amounts of proteins (50 μg) were separated on 10% and 0.275% or 15% and 0.33% (*w*/*v*) acrylamide and bisacrylamide slab gels respectively, followed by electrophoretic transfer onto nitrocellulose membranes (0.45 μm, Schleicher & Schuell, Stockbridge, GA, USA). Nitrocellulose membranes were dyed with Ponceau staining for ensuring good quality results of protein transfer and loading and subsequently they were left overnight for incubation with the appropriate antibodies (Monoclonal rabbit anti-LC3B (3868, Cell Signaling, Beverly, MA, USA), polyclonal rabbit anti-p62/SQSTM1 (5114, Cell Signaling, Beverly, MA, USA), anti-Bcl2 (7973, Abcam, Cambridge, UK) and anti-Bax (B-9) (2772, Cell Signaling, Beverly, MA, USA). Thereafter, nitrocellulose membranes were washed with TBST (3 × 5 min). Then, an 1 h incubation with a horseradish peroxidase linked secondary antibody (7074, 7076, Cell Signaling, Beverly, MA, USA) followed and membranes were washed with TBST (3 × 5 min). The blots were detected using enhanced chemiluminescence (Chemicon) on Fuji Medical X-ray film and quantified by densitometry scanning laser (GelPro Analyzer Software, GraphPad, San Diego, CA, USA).

### 2.10. Metabolomics

#### 2.10.1. Sample Preparation

Polar metabolite extraction was performed by adding 5 mg of tissue to 150 μL of an ice cold methanol:water (1:1) mixture. The tissue was then ground using a chilled mortar and pestle. The ground extract was transferred into an Eppendorf tube and subjected to ultrasonic treatment using a sonication rod for a total of 3 min, divided in six fractions of 30 s intervals separated by 2-min intervals, in ice water bath in order to avoid a significant rise of the temperature. Following sonication the extract was centrifuged at 5000 rpm for 10 min at room temperature and was then stored at −20 °C until analysis. Before analysis, each extract was allowed a brief thaw time and was then filtered throw a 20 μm syringe filter. L-Alanine-3,3,3-d_3_ was added as an injection standard to each sample to a concentration of 10 ppm before analysis.

#### 2.10.2. LC-MS/MS Analysis

Each sample was analysed on a Thermo Scientific™ TSQ Quantum™ Access MAX Triple Quadrupole Mass Spectrometer coupled to an Accela™ 1250 UHPLC pump and an Accela™ autosampler employing a Waters™ ACQUITY UPLC BEH Amide Column (1.7 µm, 2.1 × 150 mm). The applied analysis method was based on previously developed methods [74,75]. Briefly, the flow rate was set to 300 μL min^−1^. Solvent A was 95:5% Acetonitrile:H_2_O, 10 mM CH_3_COONH_4_ and solvent B was 30:70% Acetonitrile:H_2_O, 10mM CH_3_COONH_4_. A gradient elution program was applied as follows: 100% A (hold for 4 min), then to 60:40% A:B (over 21 min), then to 15:85% A:B (over 4 min and then hold for 3 min) then to 100% A (hold for 15 min). The injection volume was 5 µL.

Standards of analytical grade were purchased from Sigma-Aldrich, Alfa Aesar and Acros Organics were used for all compounds in order to verify transitions and conditions. Retention times were verified in the analytical run by injecting a global quality control standard that included all of the aforementioned compounds at a concentration of 5 μg mL^−1^. Blanks were injected after each sample in order to check for any carryover effects.

#### 2.10.3. Data Analysis and Interpretation

Sample data was analysed using Thermo ScientificTM Qual Browser, Thermo Xcalibur version 3.063. Sample comparison was performed using response ratios of the analyte peak area to the area of the injection standard. Metabolites were correlated to metabolic pathways using publicly available databases Metaboanalyst 5.0, Small Molecules Pathway Database (SMTDB) and Human Metabolome Database (HMDB).

### 2.11. Statistical Analyses

Molecular and biological analyses results were expressed as mean (± standard deviation, SD) of mean. The non-parametric Mann–Whitney U test (*p* < 0.05) was used to assess significant differences (*p* < 0.05) between control and treated specimens. Moreover, a two-level nested Anova model was applied to investigate the effect of species and tissue examined (each species, each tissue and species-tissue combination) on the parameters studied. Spearman’s rank correlation analysis was also applied for extracting intercorrelations of the parameters measured in both tissues and fish species. The above analyses were performed using the SPSS software (ver. 27, Inc. Chicago, IL, USA).

## 3. Results

### 3.1. PS-MPs Characterization

**Figure 1:**
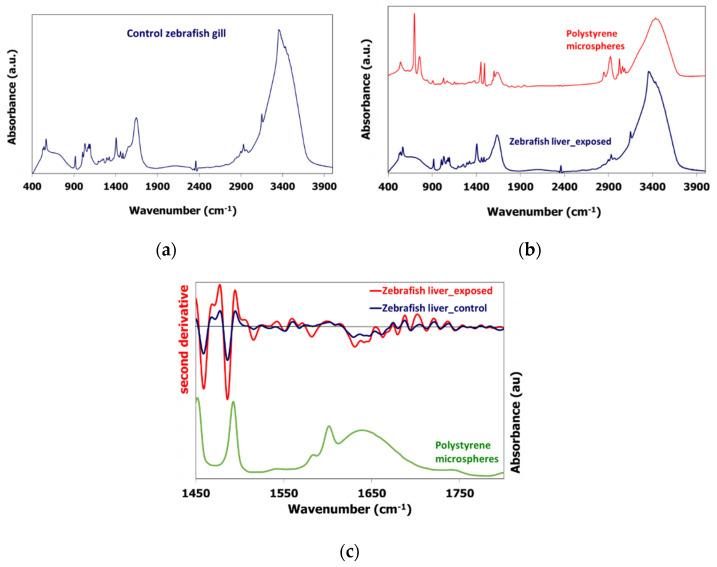
(**a**) FTIR spectrum of the control zebrafish gill, (**b**) FTIR spectrum of the exposed zebrafish liver sample and the PS microspheres, (**c**) Second derivative analysis of the exposed and control zebrafish liver samples and the absorbance spectrum of the PS microspheres.

PS-MPs are spherical with average diameter size about 8 μm and as was found in our previous work are completely amorphous [52]. During zebrafish feeding we are expecting these to be entered by food to their bodies and accumulated to several organs. This was evaluated by FTIR and micro-Raman spectroscopies. The control and exposed liver and gill samples from zebrafish and perch samples were characterized by FTIR spectroscopy. Both fish liver and gill samples exhibit similar control and exposed spectra (Figure 1, shown only spectra of zebrafish), depicting peaks corresponding to proteins; between 900 and 1300 cm^−1^ are phosphates mainly associated with RNA and DNA related nucleic acids, while in the 1300 and 1800 cm^−1^ region are protein (Amide I, II) bonds and in the 2700–3900 wavenumbers are peaks related to N–H stretching vibration of proteins [76]. The control zebrafish gill FTIR spectrum is shown in Figure 1a, while the zebrafish liver exposed samples with the PS-MPs is shown in Figure 1b. Both spectra exhibit similar peaks, while the potential PS characteristic peaks are not evident at the exposed spectra.

The PS characteristic peaks are 3025 cm^−1^ for aromatic C–H stretching vibration, C–H stretching at 2921 cm^−1^, three peaks at 1600, 1492 and 1451 cm^−1^ respectively indicates aromatic C–H bond stretching vibration and 1260, 1017, 796, 749 and 695 cm^−1^ corresponds to aromatic C–H deformation vibration. The PS-MPs characteristic peaks coincide with the control peaks, thus only high PS presence in the exposed samples would allow their spectra exhibition, as it has been reported in other FTIR studies of particles in biological media [77]. A second derivative analysis of the exposed (orange line) and control (blue line) zebrafish liver sample, which can determine minor changes in the spectra peaks, showed an increase of the peaks at 1451 and 1492 cm^−1^, as shown in Figure 1c, which can be attributed to absorbance enhancement of the exposed sample due to PS presence. PS concentration in the liver and gill samples of both zebrafish and perch species is not high enough to be evident to the primary absorbance spectra, further spectrum analysis and comparison of the exposed and control sample though can allow determining a possible limited concentration of PS.

**Figure 2:**
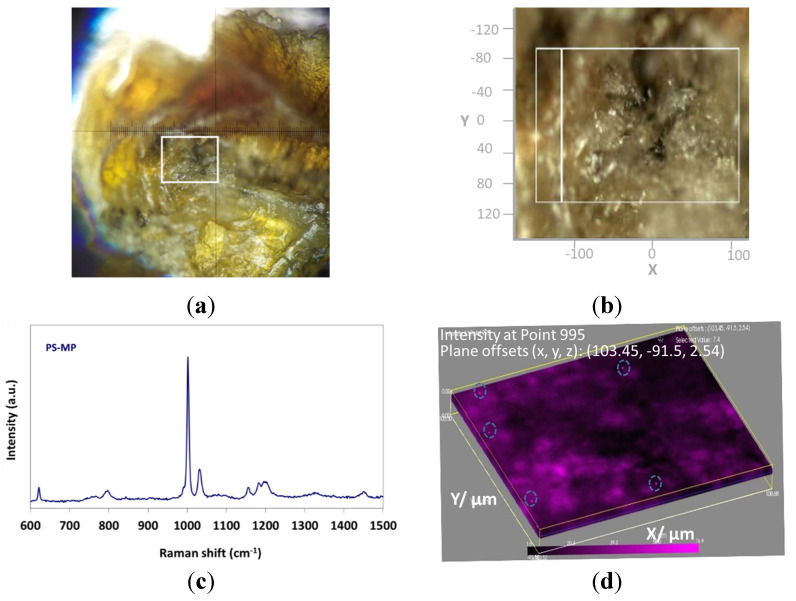
(**a**) Zebrafish gill as observed from the microscope lens, indicating the measured area with the white rectangle, (**b**) The measured area of Figure 2a in the x, y, z axes, (**c**) Raman spectrum of PS-MS, (**d**) Still image of the HR volume video map indicating the PS-MS in depth 2.54 μm of the gill area (the HR volume video map can be found in the Supplementary Material).

Even though Raman spectroscopy has been used for the characterization of MPs in zebrafish organs [78], in the current study an advanced 3D mapping characterization was performed by Raman spectroscopy detecting the polystyrene (PS) microparticles directly in the zebrafish gill without further dissolution or destruction of the organ, providing information on location, concentration and size date of the measured microplastic by an ex situ technique with micrometer resolution. An area of 255 × 192 × 10 μm of the zebrafish gill (Figure 2a) was selected for HR volume study by micro-Raman spectroscopy. The gill organic material (Figure 2b) exhibited no Raman intensity signal, while the regions where the PS-MPs were detected exhibited the characteristic PS Raman peak at 995 cm^−1^, as shown in Figure 2c. A still image of the HR volume map exhibiting the x-y plane at the depth of 2.54 μm is observed in Figure 2d; the distinct spherical pink dots are attributed to PS-MPs presence. The size of the PS-MPs was calculated from the sequential images of the z axis video movie, having a mean diameter of ~5 μm. On the current analyzed area of 490,000 μm^3^, 19 PS-MPs were detected.

### 3.2. Molecular and Biochemical Responses

#### 3.2.1. Oxidative Stress Biomarkers

**Figure 3:**
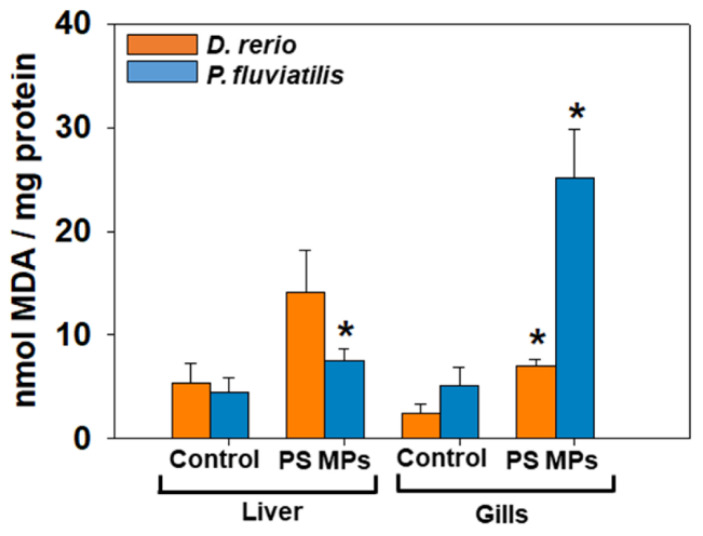
Lipid peroxidation was expressed as MDA concentrations (mean ± SD nmol mg^−1^ protein), in liver and gills of *Danio rerio* (*n* = 3 pools of 10 individuals) and *Perca fluviatilis* (*n* = 6). Mann–Whitney U test was employed to test for significance at *p* < 0.05 between all experimental groups. * denotes significant differences (*p* < 0.05) compared to the control group (*n* = 3 pools of 10 individuals and *n* = 6 for *D. rerio* and *P. fluviatilis* respectively).

Lipid peroxidation. PS-MPs caused significant increase in lipid peroxidation (which is measured by MDA increase) only in the liver of *P. fluviatilis* (*p* = 0.014, Figure 3). However, the increase observed in the gills was significant for both species (zebrafish: *p* = 0.046; perch: *p* = 0.004). Comparing the two fish response, the highest sensitivity, considering the size of response was observed in gills of *P. fluviatilis* (5 times increase for perch compared to 2.8 times for zebrafish). When comparing the tissue response in each fish, gills are more susceptible to MPs than liver in both in *D. rerio* and *P. fluviatilis* (Figure 3).

**Figure 4:**
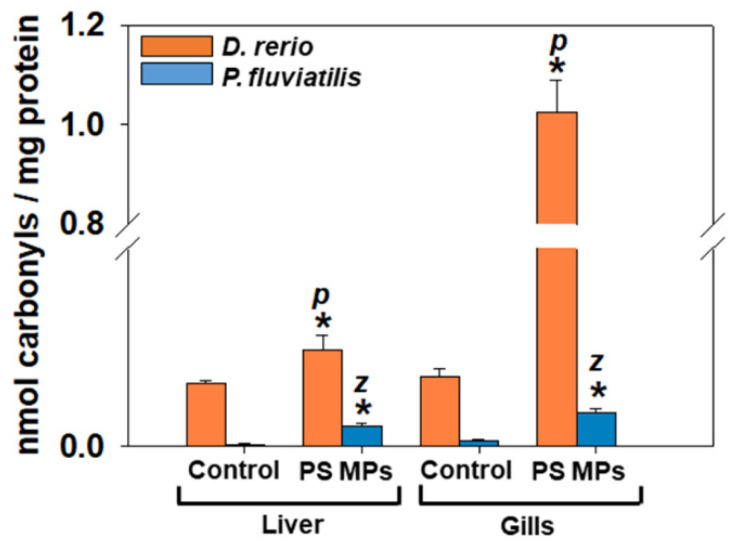
Protein carbonylation (mean ± SD nmol carbonyls mg^−1^ protein) in liver and gills of *Danio rerio* (*n* = 3 pools of 10 individuals) and *Perca fluviatilis* (*n* = 6). The results were expressed as nmol carbonyl groups mg^−1^ of protein. Mann–Whitney U test was employed to test for significance at *p* < 0.05 between all experimental groups. * denotes significant differences (*p* < 0.05) compared to the control group (*n* = 3 pools of 10 individuals and *n* = 6 for *D. rerio* and *P. fluviatilis* respectively), while *z* and *p* denote significant differences (*p* < 0.05) between *D. rerio* and *P. fluviatilis* respectively.

Protein oxidation and proteolysis (carbonyl groups and ubiquitin). Our results showed that exposure to PS-MPs revealed a significant (liver, zebrafish: *p* = 0.032; perch: *p* = 0.001; gills, zebrafish: *p* = 0.004; perch: *p* < 0.001) increase in carbonyl groups in liver and gills of both fish in relation to the respective controls (Figure 4). The highest sensitivity against MPs concerning the size of response, between the two fish was observed in the liver of *P. fluviatilis* compared to *D. rerio* (10 times and 1.5 times increase respectively compared to control) and in gills of *D. rerio* in comparison to *P. fluviatilis* (14.3 times and 5.5 times increase respectively compared to control). When comparing the tissue response in each fish, gills are more susceptible to MPs than liver in *D. rerio* and liver more sensitive than gills in *P. fluviatilis* against PS-MPs. Tissue carbonyls responses seem to be opposite than those of MDA.

**Figure 5:**
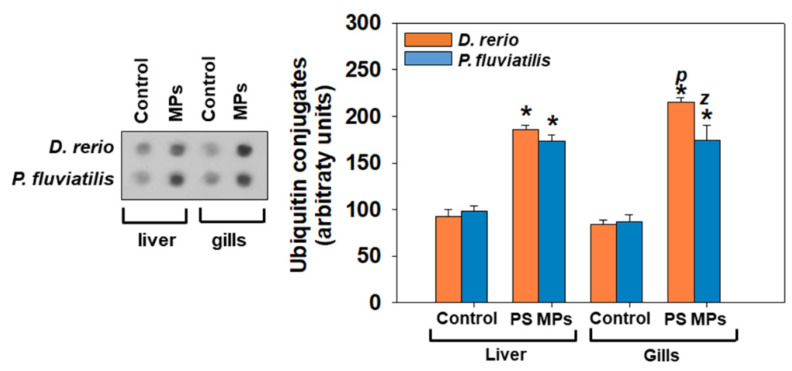
Ubiquitin conjugates (mean ± SD) in liver and gills of *Danio rerio* (*n* = 3 pools of 10 individuals) and *Perca fluviatilis* (*n* = 6). Mann–Whitney U test was employed to test for significance at *p* < 0.05 between all experimental groups. * denotes significant differences (*p* < 0.05) compared to the control group (*n* = 3 pools of 10 individuals and *n* = 6 for *D. rerio* and *P. fluviatilis* respectively), while *z* and *p* denote significant differences (*p* < 0.05) between *D. rerio* and *P. fluviatilis* respectively.

Ubiquitin conjugates in liver and gills of *D. rerio* and *P. fluviatilis* after their exposure to PS-MPs are depicted in Figure 5. In all cases ubiquitin levels were significantly (*p* < 0.05) higher in exposed animals in relation to controls.

**Figure 6:**
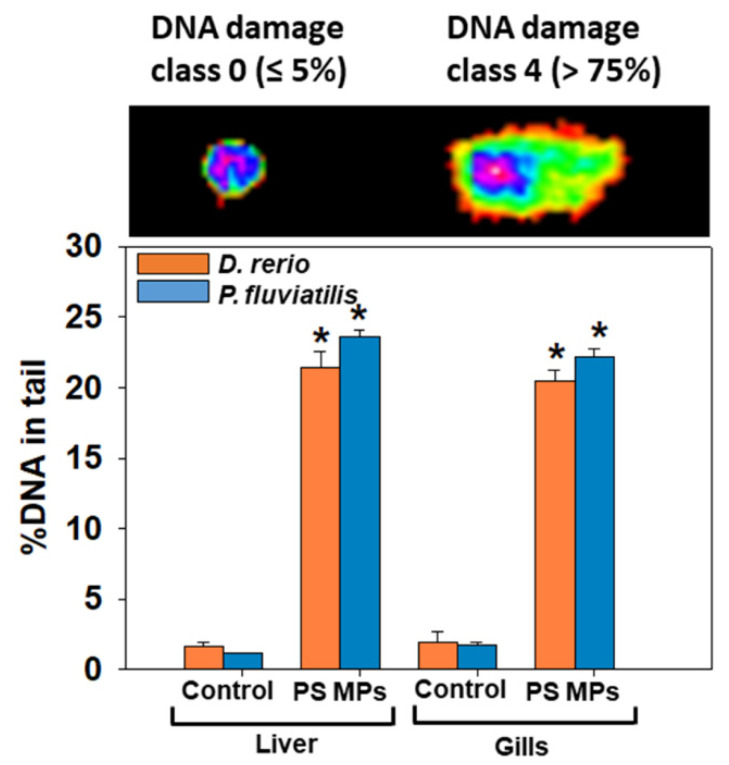
Percentage (%) of DNA damage (mean ± SD) in tail in liver and gills of *Danio rerio* (*n* = 3 pools of 10 individuals) and *Perca fluviatilis* (*n* = 6). Six slides per pool (zebrafish) and six slides per individual (perch) were measured, in order to represent technical replicates. Randomly selected 100 cells were scored from each slide (TritekCometscoreTM 1.5, TriTek Corporation, Wilmington, DE, USA). Representative pictures of DNA damage are shown. Mann–Whitney U test was employed to test for significance at *p* < 0.05 between all experimental groups. * denotes significant differences (*p* < 0.05) compared to the control group (*n* = 3 pools of 10 individuals and *n* = 6 for *D. rerio* and *P. fluviatilis* respectively). % DNA in tail and Olive moment in positive control data (1 μΜ H_2_O_2_) were 28.3 ± 5.2 and 40 ± 6.3, respectively.

Genotoxic responses (DNA damage). Our results showed that DNA in tail (%) after the exposure to PS-MPs was evident in both tissues of animals studied, revealing a significant (for liver, zebrafish: *p* = 0.009; perch: *p* = 0.004, for gills, zebrafish: *p* = 0.004; perch: *p* = 0.004) increase compared to controls (Figure 6). The response of liver and gills of both fish species regarding the increase in DNA in tail of the comets after MPs, was in the range 12 to 20 times higher in relation to their respective controls.

#### 3.2.2. Apoptosis and Autophagy

**Figure 7:**
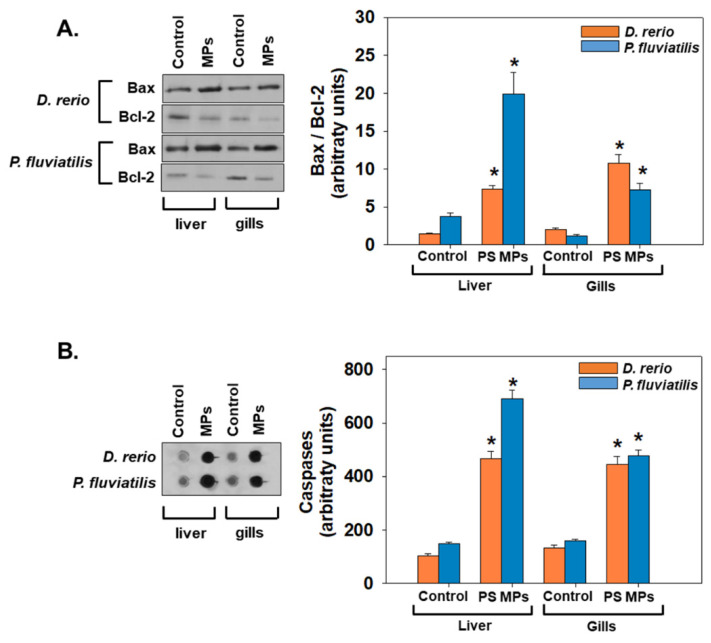
Bax/Bcl-2 ratio (**A**) and caspases levels (**B**) (mean ± SD) in liver and gills of *Danio rerio* (*n* = 3 pools of 10 individuals each pool) and *Perca fluviatilis* (*n* = 6). Tissue extracts from all groups were immunoblotted for Bax, Bcl-2 and caspases. Blots and dots were quantified using scanning densitometry. Representative blots and dots are shown. Mann–Whitney U test was employed to test for significance at *p* < 0.05 between all experimental groups. * denotes significant differences (*p* < 0.05) compared to the control group (*n* = 3 pools of 10 individuals and *n* = 6 for *D. rerio* and *P. fluviatilis* respectively).

Exposure to PS-MPs triggers apoptosis in the liver and gills of both fish species is confirmed by the increased Bax/Bcl-2 ratio and caspases levels (Figure 7). Our results showed that the ratio Bax/Bcl-2 in both tissues on both fish was increased 5 to 6 times, in comparison to the respective controls (for all cases *p* < 0.05) (Figure 7A). When comparing the two fish, exposure to PS-MPs resulted to a similar increase (5.2 times in the liver of both *D. rerio* and *P. fluviatilis*). The susceptibility of both fish against PS-MPs seems to be the same. Tissues’ responses also seem to follow the same profile against PS-MPs exposure for both fish. Caspases levels were also significantly increased in both tissues of both species compared to the control group (*p* < 0.05) (Figure 7B). Regarding tissue responses, liver seems to be more susceptible than gills in both fish studied (4.49 and 4.63 times increase in liver vs. 3.36 and 3.02 times increase in gills in zebrafish and perch, respectively).

**Figure 8:**
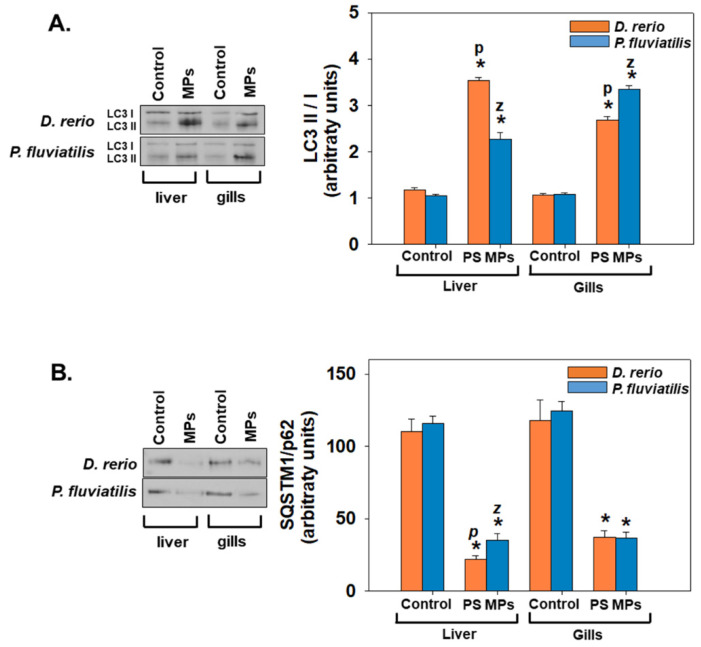
LC3II/I ratio (**A**) and SQSTM1/p62 levels (**B**) (mean ± SD) in liver and gills of *Danio rerio* (*n* = 3 pools of 10 individuals) and *Perca fluviatilis* (*n* = 6). Tissue extracts from all groups were immunoblotted for LC3II/I and SQSTM1/p62. Blots and dots were quantified using scanning densitometry. Representative blots and dots are shown. Mann–Whitney U test was employed to test for significance at *p* < 0.05 between all experimental groups. * denotes significant differences (*p* < 0.05) compared to the control group (*n* = 3 pools of 10 individuals and *n* = 6 for *D. rerio* and *P. fluviatilis* respectively), while *z* and *p* denote significant differences (*p* < 0.05) between *D. rerio* and *P. fluviatilis* respectively.

Concerning, our autophagy results, exposure to PS-MPs resulted in significant alterations (Mann–Whitney U test, *p* < 0.05) of the autophagic indicators investigated herein, confirming PS-MPs’ provoked initiation of autophagy (Figure 8). Concerning LC3 II/I ratio, PS-MPs exposure resulted in the range of 2 to 3 times increase in liver and gills of both examined fish species (for all cases *p* < 0.05) (Figure 8A). The comparison of the responses of two fish revealed similar feedback to PS-MPs. Concerning the tissue responses, the two fish responded differently, with zebrafish liver and perch gills to be more susceptible to PS-MPs than their respective controls (Figure 8A). Regarding SQSTM1/p62 levels, exposure to PS-MPs resulted to a significant (*p* < 0.05) decrease in comparison to control in both fish species, indicating the same profile of both fish against PS-MPs exposure (Figure 8B). Tissue responses also of both fish were similar (Figure 8B).

### 3.3. Inter-Species and Inter-Tissue Comparisons of Molecular and Biochemical Parameters

**Figure 9:**
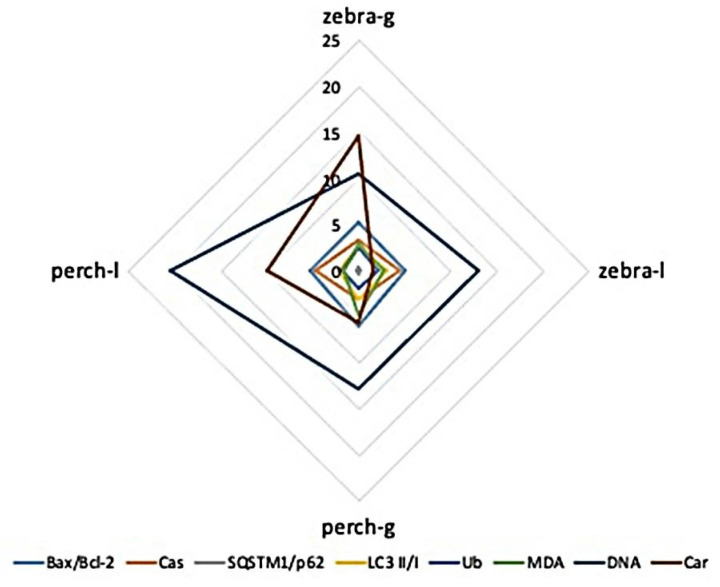
Graphical representation of molecular and biochemical biomarkers measured in the gills (g) and liver (l) of *Danio rerio* (zebrafish) and *Perca fluviatilis* (perch) (for graphical purposes caspases and ubiquitin values were divided by 10).

Regarding the sensitivity of gills in comparison to liver of each fish against PS-MPs, oxidative stress biomarkers responses, do not seem to follow similar profiles. When all molecular and biochemical parameters are examined together for assessing the response among the fish species and tissues examined, gills of both species have the highest response against 5–12 μm of PS-MPs for the majority of the parameters studied (Figure 9). Additionally, perch liver was the most liable tissue to respond to DNA damage, and zebrafish gills the most responsive to carbonyl groups (Figure 9). The results of nested Anova are presented in Table 1.

### 3.4. Correlation between Biochemical Indicators

Table 2 illustrates correlation analyses between the parameters studied in the liver and gills of *D. rerio* and *P. fluviatilis* after exposure to PS-MPs. Higher number of significant (*p* < 0.05) intercorrelations among the parameters studied were extracted in liver samples of perch and gill samples of zebrafish. It is also evident that parameter intercorrelation is independent of tissue and species, since no pattern is obviously followed (Table 2).

### 3.5. Metabolomics

In total the levels of 33 small polar metabolites were estimated which participate in pathways related mainly to amino acids, nitrogen and energy metabolism. The levels of all measured metabolites in zebrafish gills were decreased in comparison to the control, which is probably associated with reduced metabolic rate in gills as shown in Table 3. The levels of L-phenylalanine, L-carnitine and L-proline exhibited greatest decrease by 93, 91 and 91%, respectively, whereas salicylic acid, L-lactic acid and choline were least affect exhibiting a decrease of 30, 44 and 49%, respectively. Similarly, in exposed perch gills the levels of all metabolites decreased except salicylic acid and L-phenylalanine that were increased by 210 and 64%, respectively whereas the metabolites whose levels exhibited the greatest decrease were acetyl-L-carnitine (ALCAR), L-alanine, L-glutamic and pyruvic acid, 96, 86, 86 and 75%, respectively.

In zebrafish livers the greatest perturbations were observed among nucleic acids metabolites; adenine and adenosine exhibited significant increases, 185 and 127% respectively, while the greatest decreases were exhibited in hypoxanthine (69%), uridine (61%) and deoxyadenosine levels (55%). Hypoxanthine is also a known marker of exercise exhaustion [79]. Among amino acids L-valine, L-arginine, L-phenylalanine, L-asparagine and L-proline exhibited a decrease of almost 50% or greater while L-glutamine is the only amino acid that exhibited increase greater than 50%. On the other hand L-arginine, succinic acid and adenosine levels in perch liver were increased by 221, 120 and 120%, respectively, while 2-oxoglutaric acid, hypoxanthine, citrulline, L-creatinine, ALCAR and adenine decreased to levels less than half compared to the control.

The results of nested analysis applied to nine metabolites measured in gills and liver tissue of both species are presented in Table 4. Only L-glutamic acid exhibited significant differences when species or tissue factor are considered while no significant difference was observed when species-tissue combination is examined. However, the effect of species-tissue was significant to all other metabolites examined (Table 4).

## 4. Discussion

In the present study, PS-MPs’ effects were studied by measuring and comparing the responses of biochemical and molecular parameters as well as metabolite levels in the gills and liver of two freshwater fish species, zebrafish, *D. rerio* and perch, *P. fluviatilis*. Fish were fed with food supplemented with PS-MPs with particle size 5–12 μm, at concentrations close to those estimated for each species by the EC_50_ value by in vivo experiments. The concentrations of PS-MPs that each fish was treated were close to the physiological tolerance of each fish to PS-MPs effect. Our outcomes revealed that exposure of both fish species to PS-MPs at sublethal concentrations caused toxic effects on fish tissues after 21 days of exposure. Accordingly, the metabolic pathway analysis revealed that PS-MPs concentrations caused a significant effect to the metabolism of glycerolipid, the unsaturated fatty acids biosynthesis as well as the gluconeogenesis ability of studied fishes. The difference in the response against PS-MPs of the two species could be attributed to the difference in weight (100 times greater), length (four times greater) and lifespan (four times greater) of the examined fish.

### 4.1. Oxidative Stress

The exposure of fish to a number of pollutants including nanoparticles, biomaterials as well as MPs causes oxidative stress, due to ROS overproduction [44,80]. Subsequently ROS promote protein and lipid peroxidation as well as genotoxic damages [46,47,81,82]. ΜDA is the principal biomarker of lipid peroxidation that is significantly increased in both fish tissues; MDA has already been proved to be a reliable biomarker for aquatic animals and terrestrial snails [46,47,52,82].

Another consequence due to MPs fish exposure is the alteration of cellular proteins [83,84]. Proteins that are carbonylated at a high degree are considered to be dysfunctional, are gathered as a mass of proteins, linked with a covalent bond and cannot be proteolyzed, causing several detrimental effects on the cell functions [85]. Protein carbonylation has been proposed as a sensitive indicator of protein oxidation in zebrafish, Prussian carp and terrestrial snail [46,47,86]. Wen et al. [87] report significantly increased carbonyl levels in liver of the fish *Symphysodon aequifasciatus* after exposure to three concentrations of MPs (0, 50 and 500 μg L^−1^) together with two levels of Cd (0 and 50 μg L^−1^) for 30 days. Thus, it may be assumed that these diffusible products of molecular peroxidation originate from the reaction catalyzed by myeloperoxidase that produces potent oxidants, causing cell oxidative injury which successively, may produce deleterious effects on fish organism.

Oxidative stress apart from causing peroxidation of lipids and protein oxidation also produces DNA damage in fish tissues [46]. In particular, ROS can lead to several DNA modifications. These include: bases degradation, breaks of single- or double-stranded DNA, sugar-bound, purine or pyrimidine modifications, mutations which can include translocations or deletions, and finally cross-linking with proteins [88]. Our results exhibited a significant increase in DNA damage in both fish tissues, with perch liver exhibiting the highest response. Similar to our results, Pannetier et al. [89] reported oxidative DNA damage to a liver cell line of *Oryzias latipes* larvae fed for 30 days with three doses of MPs (0.01, 0.1 and 1% *w*/*w* in fish food). Moreover, bivalves which were exposed to polyethylene (PE) and polystyrene (PS) microplastics, sized 1000–100 μm, at concentrations of 0.5, 5 and 50 μg L^−1^ [90] or to PS-MPs at 1 mg L^−1^ (20 μm) concentration for 14 days, [90,91] showed irreversible loss of DNA integrity.

Regarding the sensitivity of gills in comparison to liver of each fish against PS-MPs, MDA, protein carbonyls and DNA damage responses, do not seem to follow similar profiles. Wang et al. [38] reported higher MDA levels in the liver rather than in the gills of the marine medaka (*Oryzias melastigma*) after exposure to 2, 20 and 200 μg L^−1^ concentrations of 10 μm PS-MPs for 60 days. Furthermore, our results showed that tissue carbonyls responses seem to be opposite than those of MDA. Yang et al. [92] revealed lipid peroxidation and oxidative stress in zebrafish larvae when exposed to either 6:2 chlorinated polyfluorinated either sulfonate (F–53B), 50 ng mL^−1^ polystyrene microplastics (PS-MPs) or their combination for 7 days. Similar inflammatory responses were reported after long term (more than 90 days) treatment with diet enriched with 10% PS-MPs in the intestine of *Sparus aurata* [38,65,88]. In addition, regarding MPs effect on *Sparus aurata* liver, although no change in MDA levels was observed, increased protein damage due to low-density polyethylene MPs (size between 100 and 500 μM) exposure for 90 days has been reported, that was attributed to an increase in myeloperoxidase (MPO) activity, indicating an inflammatory response [93]. These results as well as other studies [39,94,95], suggest that MPs ingestion provoke the antioxidant defense; however, this was not enough for the prevention of oxidative damage.

Protein degradation, which can be a consequence of ribosomal dysfunction and/or disrupted structure [96], is the vital intracellular task that accounts both for housekeeping as well as for the management of various functions of the cell, including that of dealing with different types of stress [97]. Proteasomes and lysosomes constitute the most important proteolytic systems. Any change in these proteolytic systems affects many metabolic pathways of the cell. Proteolysis of different proteins of the cell is achieved by the ubiquitin–proteasome system (UPS) [98]. Furthermore, the ubiquitin–proteasome pathway (UPP), which is also activated by oxidative stress [99], holds a major part in multitude functions of the cell as DNA repair, signal transduction, as well as dealing with different types of stress, e.g., oxidation, exposure to heavy metals [97]. Our results showed significantly higher ubiquitination in the liver and the gills of both fish examined, with differences in the size of response of each tissue as well as between the two fish species. In precise, gills had a higher response compared to liver tissue, while when the two fish species compared, zebrafish seems to respond with higher sensitivity than *P. fluviatilis*. Thus, our results encourage the suggestion of ubiquitin as a biomarker against PS-MPs, as has already been proposed for other pollutants in fish [46,47].

### 4.2. Molecular Events, Apoptosis and Autophagy

Various stimuli, including ROS generation and the subsequent oxidative stress [100,101], can trigger multiple signaling pathways, which are responsible for a cell’s fate. Apoptosis, which is triggered either by inside boosts, or by extracellular impulses, is probably the most prominent cell death mechanism. Caspase 3 is involved in the innate immune system and apoptosis, in order to protect the fish when it is under stress-induced toxicity [102]. Caspase activity has been found to be a valuable biomarker for the detection of stress-induced apoptosis in fish [46,47,52,103]. In addition, the ratio of Bax/Bcl2 reflects the activation of pro-caspase and occurrence of apoptosis [104].

Under certain circumstances, while autophagy presents a different mechanism, it can also lead to cell death. Autophagy’s key role and function is the survival of the cell. Autophagy also is an adaptive response under stressful conditions [105]. Given that the life of a cell is at stake, there is molecular crosstalk between apoptosis and autophagy pathways. The nature of these interconnections is diverse and ranges from protein–protein interactions and post-translational modifications via the deterioration of molecular components by distinct proteins and organelles [106]. Excess cellular levels of ROS which lead to damage to proteins, nucleic acids, lipids, membranes and organelles, trigger cell death processes such as apoptosis [100]. Moreover, in recent years, a growing amount of evidence argues for ROS being among the main intracellular signal transducers sustaining autophagy [101]. Thus, ROS participates in the interplay between autophagy and apoptosis by its ability to mediate the redox signaling pathways. However, the molecular machinery linking autophagy to apoptosis is still being elucidated.

Microtubule-associated proteins light chain 3 (LC3) are autophagy pathway’s principal proteins where they serve in selecting the autophagy substrate and autophagosome biogenesis. LC3 is the most extensively used indicator of autophagosomes [107]. In addition, Sequestosome-1 (SQSTM1), which is the ubiquitin-binding protein p62, is a protein of the autophagosome cargo which marks other proteins for discriminatory autophagy. In the process of autophagy SQSTM1 is degraded. Both the ratio LC3II/I, as well as SQSTM1 are widely used as indicators of autophagy [52,108,109,110,111,112]. The significant elevation of apoptosis and autophagy markers recorded in our study denotes a parallel increase of apoptosis together with autophagy generally in the tissues of both fish studied; however, no clear pattern is evident for the intertissue and interspecies differences. Accordingly, when several marine organisms are exposed to MPs, increased levels of apoptosis are observed. In specific, key genes’ related to Casp3 and Tp53 transcriptional changes were increased after exposure of sheepshead minnow to polyethylene MPs microspheres with diameters 150–180 μm [113]. Moreover, exposure of adult zebrafish to two concentrations of high-density (100 and 1000 μg L^−1^) polyethylene and polystyrene microplastics for twenty days [114] and *Mytilus edulis* exposure to high density polyethylene nonuniformly shaped grains ranging >0–80 μm in size [115] has resulted in apoptosis activation in their tissues. Similarly, concerning autophagy, a significant presence of autophagy vacuoles was observed in the enterocytes of planarians *Dugesia japonica* fed with polyethylene microsphere mixtures with a diameter ranging from 1 to 10 μm or 10–27 μm sized plastic particles [116]. Except for marine organisms and mammals, exposure to microparticles/plastics and specifically PS-MPs has led to apoptotic or autophagic cell death in human cell lines such as gastric cancer cells (AGS) after exposure to 500 nm and 60 nm polystyrene nanoplastics at concentrations 1, 5, 10, 50, and 100 mg L^−1^ [117] and macrophages [118].

### 4.3. Metabolomics

To our knowledge this is the first study on MPs’ effects of on the metabolome of gills in fish and only the second on fish liver. In accordance with all previous studies MPs affected cellular function and metabolism in all tissues tested. Lu et al. [119] using NMR in order to study the effects of PS-MPs on the metabolome of zebrafish liver, reported that the metabolic profile was altered significantly predominantly disturbing the lipid and energy metabolism. They also showed that MPs size and concentration may be correlated with different alterations in the metabolome. Recently, Dimitriadi et al. [52] studied the effects of PS-MPs on metabolites of the heart tissue of zebrafish demonstrating similarly that metabolites related to amino acid and energy metabolism exhibited significant decrease. Teng et al. [120] reported metabolic alterations and inflammatory responses in the whole oyster *Crassostrea gigas* after exposure to irregular MPs composed of polyethylene and polyethylene terephthalate at concentrations of 10 and 1000 μg L^−1^ for 21 days. Qiao et al. [121] demonstrated that when zebrafish were exposed to PS-MPs (5-μm beads; 50 μg L^−1^ and 500 μg L^−1^) for 21 days, their gut exhibited metabolome and microbiome responses, oxidative stress and inflammation. Other studies that examined the effects of polyethylene MPs on larval zebrafish after exposure to 1 to 4 μm at concentrations of 0, 10, 100, and 1000 μg L^−1^ for 7 days [122,123] and in developing zebrafish when exposed to 0.02 to 200 mg L^−1^ concentrations of MPs, sized 65 nm and 20 μm for 7 days [124], also demonstrated alterations in energy, glycolipid and lipid metabolism as well as in the microbiome of the fish.

### 4.4. Interspecies and Intertissue Comparisons

Based on the intercorrelation results of the molecular and biochemical parameters studied in both fish tissues it may be deducted that MPs toxicity mechanism is species and tissue specific. Moreover, the results of nested Anova indicated significant relations of almost all (with the exception of Bax/Bcl-2 ratio) molecular and biochemical parameters studied as well as of the metabolites (with the exception of L-glutamic acid) when species- tissue is considered as the model design. However, since the two fish species differ in size, the ability of the MPs to be translocated may be different, so thus differences in the fish responses are expected. According to our results, the tissues response between the two different organisms did not follow a similar profile as also shown for several fish species [125,126]. This could be indicative of relatively closely related toxicity mechanisms in the livers despite the differences in life span and size and the fact that toxicity was earlier shown to be species and tissue specific [46,47]. Interestingly liver is known as the major detoxifying organ in all organisms and in the present study it suffered greater DNA damage in both species than gills.

Our group has recently demonstrated PS-MPs effects also on the heart tissue and the whole fish, in specific showing frequency reduction of ventricular heart contraction, decrease of swimming velocity and internalization of the MPs in the heart of *D. rerio* [52]. Thus, it is becoming evident that PS-MPs pollution at sublethal concentrations impacts most essential organs in a mechanism that involves oxidative stress, inflammation and metabolic alterations.

### 4.5. Internalization-Toxicity Induction by PS-MPs

The literature concerning absorption mechanism and MPs accumulation in marine and freshwater fish is limited. It has been reported for nanoparticles that several significant parameters define their absorption rates: size, aggregation, distribution, and cell sedimentation. Endocytosis, phagocytosis or pinocytosis can facilitate absorption [55,127]. In specific it has been reported that PS and polycarbonate nanoplastic particles are internalized through phagocytosis by neutrophils in the kidneys of the fathead minnow (*Pimephales promelas*) [128,129]. Kashiwada [130] detected 39 nm PS particles in liver, intestine and gonads of the medaka *Oryzias latipes*, which most possibly entered in gills and/or gut epithelium and were transported through the bloodstream. In particular for zebrafish, detection of 5-µm and 20-µm sized MPs was observed in the liver, gut and gills. While in some cases, MPs/NPs were accumulated in the gut of larvae or adult individuals, in other cases they are found in gill and liver [131].

In the present study, the exposed groups of treated with PS-MPs fish showed statistically significant variations from the control group, in all the examined parameters. Therefore, in line with the responses of the parameters measured, our results indicate a toxic impact PS-MPs exert on the liver and gill cells of both fish, with specific biomarkers responded greater either in gills or in liver, while DNA damage was experienced greater in liver tissue of both species than gills. Towards in understanding our findings, in relation to the increase in all the oxidative stress biomarkers, the change in apoptotic and autophagic markers, as well as the change in metabolite profile, we rely on the fact that in general the animal exposure to certain exogenous effectors, as MPs, provoke ROS production, inflammation and immune system changes [44,80,111].

In fact, according to the latter data, MPs especially of 5–12 μm sized, as those used in the present study, may be internalized from the food to the gut and then transferred via blood to gills and liver of fish. Uptake of MPs in liver and gills has been shown by the present study’s results. MPs’ high surface area could cause ROS production in the tissues leading to oxidative stress [113]. This increased oxidative stress provokes the increase of ROS production that subsequently leads to peroxidation of lipids and protein carbonyls together with increase in DNA damage [132]. Moreover, cellular components and MPs interaction can influence cell signaling, thus causing activation of proteolysis, apoptosis and autophagy processes. In parallel to the activation of all the latter events in fish tissues metabolic alterations as a result of oxidative stress also occurs. These results indicate that MPs’ accumulation and distribution in both fish gills and liver dramatically influence tissues toxicity. Thus, our results reveal that PS-MPs by generating oxidative stress, alter the functionality and metabolism of liver and gills of freshwater fish, and finally affecting the fish fitness for survival.

## 5. Conclusions

The outcomes of the present study indicate that cellular components and PS-MPs interaction produce a toxic impact by generating oxidative stress on the liver and gills of both fish species studied, as shown by lipid peroxidation, protein oxidation and DNA damage measurements. In parallel, cell signaling is influenced, thus provoking molecular inductions as apoptosis, ubiquitylation, autophagy and metabolic alterations affecting mainly amino acids, nitrogen and energy metabolism. The levels of most of the metabolites in both fish tissues were reduced in comparison to the control, which is probably associated with reduced metabolic rate after PS-MPs treatment.

In general, toxicity response was species and tissue specific with each biomarker showing different responses in gills and liver. Among biochemical indices DNA damage exhibited greater response in the liver of both species compared to gills. The alterations of metabolites in gills were more profound to those observed in liver. MDA, protein carbonylation, DNA damage, ubiquitin levels, caspases, Bax/Bcl-2 ratio, LC3 II/I and SQSTM1, as well as metabolites profile continue to provide essential information on cellular functionality in biomonitoring studies against PS-MPs in freshwater fish.

In addition, our results showed that *P. fluviatilis* seems to be more liable to respond against PS-MPs compared to *D. rerio*, at the experiment’s conditions.

MPs constitute an increasing environmental hazard and have been shown to affect most organs in aquatic organisms at the cellular, metabolic and functional level. The current findings provide data that promote our understanding of the interplay of the effects between tissues in fish species that may eventually lead to the selection of appropriate biomarkers for MPs pollution, food safety and fishing stocks sustainability.

## Figures and Tables

**Table 1:** Nested Anova of the effects of species, tissue and species-tissue combination on the molecular and biochemical indicators studied.

Parameter	Species	Tissue	Species -Tissue
MDA	ns	ns	F(1,24) = 7.14, *p* = 0.013
Car	ns	ns	F(1,24) = 36.8, *p* = 0.001
Ub	ns	ns	F(1,24) = 7.74, *p* = 0.010
DNA	ns	ns	F(1,24) = 7.67, *p* = 0.011
Bax/Bcl-2	ns	ns	ns
Cas	ns	ns	F(1,24) = 57.86, *p* = 0.000
LC3 II/I	ns	ns	F(1,24) = 144.50, *p* = 0.000

ns = *p* > 0.05.

**Table 2:** Spearman’s rank correlation matrix between molecular and biochemical biomarkers measured in the gills and liver of *Danio rerio* and *Perca fluviatilis*.

	*Danio rerio*								*Perca fluviatilis*							
	DNA	MDA	Car	Ub	LC3 II/I	Bax/Bcl-2	SQSTM1/p62	Cas	DNA	MDA	Car	Ub	LC3 II/I	Bax/Bcl-2	SQSTM1/p62	Cas
DNA					−											
MDA				+	+	+										
Car		+			+					−						
Ub						+										
LC3 II/I						+						+			+	
Bax/Bcl-3					+							+	+			−
SQSTM1/p62				−								−	−	−		
Cas				+			−					−	−	−	+	
liver	gills	(+) possitive		(−) negative		*p* < 0.05										

**Table 3:** Alterations of metabolites levels in gills and liver of exposed fish in comparison to the control, expressed as %.

	Gills		Liver	
	Zebrafish	Perch	Zebrafish	Perch
L-Asparagine	−78		99	61
L-Glutamine			−36	−15
L-Glutamic acid	−83	−86	−8	−21
L-Valine	−79	−63	194	21
L-Lysine	−72		−23	−14
L-Alanine	−83	−86	55	−30
L-Proline	−91	−63	96	8
L-Tyrosine	−84	3	67	45
L-Phenylalanine	−93	64	113	67
L-Arginine	−51		135	221
Ornithine			−15	−5
Citrulline	−67		88	−58
Creatine			59	−34
Creatinine			62	−57
Pyruvic acid		−75		
L-Lactic acid	−44	−66		
Succinic acid	−56	−54	20	120
2-Oxoglutaric acid			−31	−61
L-Carnitine	−91	−61	96	50
ALCAR	−80	−96	−55	−56
Butyric acid			28	28
Hypoxanthine			223	−59
Adenine			−56	−54
Adenosine			−65	120
Deoxyadenosine			124	−44
Uridine			155	−45
Salicylic acid	−30	210	−51	7
Betaine	−70	−45	−11	75
Choline	−49	−33	94	8
Putrescine		−43		
Niacinamide	−71	−59	7	17
Riboflavin				1
Trehalose			−74	−81

**Table 4:** Nested Anova of the effects of species, tissue and species-tissue combination on the metabolites studied.

Metabolites	Species	Tissue	Species -Tissue
Betaine	ns	ns	F(1,8) = 124.09, *p* = 0.000
Choline	ns	ns	F(1,8) = 270.74, *p* = 0.000
L-alanine	ns	ns	F(1,8) = 23.83, *p* = 0.001
L-carnitine	ns	ns	F(1,8) = 154.40, *p* = 0.000
L-glutamic acid	F(1,1) = 6384.34, *p* = 0.008	F(1,1) = 531,344, *p* = 0.001	ns
L-phenylalanine	ns	ns	F(1,8) = 139.07, *p* = 0.000
L-proline	ns	ns	F(1,8) = 14.61, *p* = 0.005
Salicylic acid	ns	ns	F(1,8) = 380.88, *p* = 0.000
Succinic acid	ns	ns	F(1,8) = 4.75, *p* = 0.061

ns = *p* > 0.05.

## Supplementary Materials

The following are available online at https://www.mdpi.com/article/10.3390/toxics9110289/s1.
