# Supplementary material for: Toxicity and Functional Tissue Responses of Two Freshwater Fish after Exposure to Polystyrene Microplastics
Source: Toxics. 2021 Nov 2;9(11):289. doi: 10.3390/toxics9110289 (PMC8625933; doi:10.3390/toxics9110289)
Supplement: Supplementary file 1 [file toxics-09-00289-s001.zip › toxics-1388642-supplementary.pdf]

toxics MDPI

Supplementary Materials: Toxicity and Functional Tissue Responses of Two Freshwater Fish after Exposure to Polystyrene Microplastics

Martha Kaloyianni [1], Dimitra C. Bobori [2,*], Despoina Xanthopoulou [1,2,†], Glykeria Malioufa [1,†], Ioannis Sampsonidis [3], Stavros Kalogiannis [3], Konstantinos Feidantsis [1], Georgia Kastrinaki [4], Anastasia Dimitriadi [5], George Koumoundouros [5], Dimitra A. Lambropoulou [6], George Z. Kyzas [7] and Dimitrios N. Bikiaris [8,*]

1. Synthesis of PS-MPs

For the preparation of microparticles, firstly a 2% w/v PS (CAS No. 9003-53-6, Batch No. 09616AC, Mw = 230,000, Aldrich®, USA) solution was prepared in CHCl3 (Merck, Germany) at room temperature. An aqueous poly(vinyl alcohol) (PVA) (87–89%, CAS No. 9002–89–5, Lot No. MKCF7294, Mw = 13,000–23,000, Aldrich®, USA) solution was also prepared of 1 % w/v, after 1 h of magnetic stirring at 80 °C. Then, the polymer solution (5 mL of volume parts) was added with pipette Pasteur in well-separated drops into the aqueous solution (50 mL of volume intervals) and intensive mechanic stirring followed for 2 min (Ultra-Turrax®, T10 basic, IKA®, Germany) to all directions. The emulsion produced was left to be stirred magnetically for 24 h at ambient conditions to make sure that the organic solvent has been evaporated. Thus, the microparticles are dispersed into the aqueous environment and are collected by double centrifugation (Unicen 21, CE 126, Orto Alresa, Spain) at 4,000 rpm for 20 min each at room temperature. The white precipitation of PS microparticles is washed and gathered with deionized H2O and dried thereafter at 80 °C for 24h under vacuum.
